# The Chronic Effect of Transgenic Maize Line with *mCry1Ac* or *maroACC* Gene on Ileal Microbiota Using a Hen Model

**DOI:** 10.3390/microorganisms7030092

**Published:** 2019-03-24

**Authors:** Liang Chen, Ruqing Zhong, Lilan Zhang, Hongfu Zhang

**Affiliations:** State Key Laboratory of Animal Nutrition, Institute of Animal Science, Chinese Academy of Agricultural Sciences, Beijing 100193, China; shengji0202@163.com (L.C.); ruqing_zhong@163.com (R.Z.); zhanglilan92@163.com (L.Z.)

**Keywords:** transgenic, maize, ileal microbiota, safety

## Abstract

The experiment was to determine the chronic effects of two transgenic maize lines that contained the *mCry1Ac* gene from the *Bacillus thuringiensis* strain (BT) and the *maroACC* gene from *Agrobacterium tumefaciens* strain (CC), respectively, on ileal microbiota of laying hens. Seventy-two laying hens were randomly assigned to one of the three dietary treatments for 12 weeks, as follows: (1) nontransgenic near-isoline maize-based diet (CT diet), (2) BT maize-based diet (BT diet), and (3) CC maize-based diet (CC diet). Ileum histological examination did not indicate a chronic effect of two transgenic maize diets. Few differences were observed in any bacterial taxa among the treatments that used high-throughput 16S rRNA gene sequencing. The only differences that were observed for bacterial genera were that *Bifidobacterium* belong within the *Bifidobacteriaceae* family tended to be greater (*p* = 0.114) abundant in hens fed the transgenic maize-based diet than in hens fed the CT diet. Birds that consumed the CC maize diet tended to have less abundance (*p* = 0.135) of *Enterobacteriaceae* family in the ileum than those that consumed the CT maize diet. These results indicate the lack of adverse effects of the BT maize and the CC maize lines on the ileal microbiota of hens for long term and provide important data regarding biosafety assessment of the transgenic maize lines.

## 1. Introduction

Consumers are becoming increasingly aware of the important effect of certain food on the intestinal microbiota [1], because a strong relationship between intestinal microbiota and host health is found in the recent analysis of the intestinal microbiome [2,3]. Especially, the controversy regarding safety for increasing usage of genetically modified (GM) crops in the world remains to be resolved [4,5,6]. Therefore, safety assessment in relation to the effect of GM food and feed on intestinal microbiota is very important. In fact, the effects of GM food and feed on the host bacterial populations have been recommended to be included in the guidelines of European Food Safety Authority [7].

Transgenic maize was planted with 59.7 million hectares and it accounted for 31% of the global maize production in 2017 [8]. Most of the GM maize that is cultivated in the world is insect resistant, herbicide tolerance, or a combination of both traits. Two transgenic maizes are currently under development in China [9]. A transgenic maize line was produced by the insertion of the *mCry1Ac* gene that was derived from *Bacillus thuringiensis* strain (BT) and transcription of the *mCry1Ac* gene confers resistance to insect damage [9]. Another transgenic maize line was produced by the insertion of the *maroACC* gene derived from the *Agrobacterium tumefaciens* strain (CC) and a gene shuffling process to optimize the kinetics of glyphosate acetyltransferase activity for acetylating the herbicide glyphosate functionally improved the *maroACC* gene [9]. The development of transgenic maize provides growers with such benefits [10], but its presence of food and feed has been the focus of attention that is related to potential health risks.

No completely consistent effects of the BT maize-based diet on intestinal bacteria of animals were found. Feeding the BT maize-based diet to sheep for 36 months using the bacterial culture methods did not affect the ruminal microbiota [11]. Using real-time PCR analysis or 16S rRNA gene sequencing, short-term feeding the BT maize did not affected ruminal bacterial communities of cows [12,13,14]. Although few differences in the compositions of the cecal microbiotas of pigs that were fed the BT maize diet for 31 days were observed using 16S rRNA gene sequencing, a high cecal abundance of *Enterococcaceae*, *Erysipelotrichaceae,* and *Bifidobacterium,* and a low abundance of *Blautia* were reported [15]. Buzoianu et al. [16] also found high abundance of fecal *Firmicutes* of offspring and a low abundance of fecal *Proteobacteria* of sows and offspring at weaning fed the BT maize diet.

To date, research investigating the effect of feeding GM food and feed on gastrointestinal bacterial communities has been limited to studies in large intestinal and fecal microbiota. The microbiota in the small intestine also has a very important effect on immune response and metabolic and endocrine functions [17], however, little information is obtained for the effect of transgenic maize on small intestinal microbiota. Therefore, the objective in the present study was to determine the chronic effect of feeding a BT maize-based diet and a CC maize-based diet for 12 weeks on ileal microbiota using the pure-line White Leghorn hen as a model.

## 2. Materials and Methods

### 2.1. Animals and Experimental Design

The Institutional Animal Care and Use Committee of the Institute of Animal Sciences at the Chinese Academy of Agricultural Science (Beijing, China) reviewed and approved by the experimental protocol. A total of 72 pure-line White Leghorn hens (55-week old; National Engineering Laboratory for Animal Breeding, China Agricultural University, Beijing, China) were randomly assigned into one of three dietary treatments: (1) nontransgenic near-isoline maize-based diet (CT diet); (2) BT maize-based diet (BT diet); and, (3) CC maize-based diet (CC diet). Each treatment was fed to eight cages of birds (replicates) and three birds per replicates. The birds were housed in three-tier battery cages and were kept with ad libitum access to feed and water. The temperature was maintained at 22 ± 3 °C for a light cycle of 16 h light/8 h dark. All of the birds were kept healthy during the study period.

### 2.2. Maize and Diets

The isogenic maize, the BT maize, and the CC maize were simultaneously grown. All of the diets were formulated to meet or exceed the nutrient requirements for poultry (NRC, 1994; Ministry of Agriculture of P. R. China, 2004) as a guideline (Table S1). Maize ingredients and experimental diets samples were measured for the proximate composition (dry matter, ether extract, crude ash, crude protein, amino acid, total calcium, and total phosphorus) [18,19,20]. The crude protein was calculated by estimating nitrogen content using the combustion method and multiplying with a factor 6.25 (FP2000 nitrogen analyzer, Leco Corp., St. Joseph, MI, USA). The gross energy concentration of ingredients and diets was determined while employing an adiabatic bomb calorimeter (Model 6400, Parr Instruments, Moline, IL, USA). The diets and ingredients were also analyzed for acid detergent fiber and neutral detergent fiber [21]. Starch was determined using the Megazyme Total Starch Assay Procedure based on thermostable α-amylase and amyloglucosidase (Megazyme International Ireland Ltd., Wicklow, Ireland).

### 2.3. Organ Sampling and Histological Analysis

One hen per pen (eight hens/treatment) was humanely euthanized at the end of 12 weeks and a gross necropsy was performed. Ileal tissue sampling and the determination of villus height, crypt depth, villus height/crypt depth ratio, and number of goblet cells per villus and per millimeter villus were performed. Ileal content was quick collected from each bird (four hens/treatment).

### 2.4. DNA Extraction and PCR Amplification

All of the ileal digesta samples were frozen on liquid nitrogen immediately after collection and stored at −70 °C until processed for DNA extraction. Total DNA was extracted from individual digesta samples using a QIAamp DNA stool mini kit (Qiagen, Hilden, Germany), according to the manufacturer’s instructions and quantified using a NanoDrop 1000 spectrophotometer (Thermo Scientific Inc., Wilmington, DE, USA) [22]. The V3 and V4 regions of the bacterial 16S rRNA gene were PCR amplified from ileal DNA extracts while using bacterial universal primer 338F and 806R with an eight-base sequence unique to each sample as a barcode [23,24]. Each PCR contained 10 ng template DNA, 0.8 μL forward primer (5 μM), 0.8 μL reverse primer (5 μM), 0.4 μL FastPfu Polymerase, 2 μL of 2.5 mM dNTPs, and 4 μL of 5× FastPfu buffer in a total volume of 20 μL. The PCR cycle was carried out as follows: denatured by 95 °C for 3 min, followed by 27 cycles of 95 °C for 30 s, 55 °C for 30 s, and 72 °C for 45 s, then 72 °C for 10 min, and held at 4°C. All of the PCR amplifications were performed in an ABI GeneAmp^®^ 9700 (Applied Biosystems, Foster City, CA, USA).

### 2.5. Illumina miseq Sequencing and Bioinformatics Analysis

Visualization under UV light following electrophoresis in a 2.0% agarose gel verified the presence of the target amplicons. Amplicons were purified using the AxyPrep DNA Gel Extraction Kit (Axygen Biosciences, Union City, CA, USA) according to the manufacturer’s instructions and quantified using QuantiFluor**™**-ST (Promega Corporation, Madison, WI, USA). The puri**fi**ed amplicons were pooled in equimolar and paired-end sequence (2 × 250) on an Illumina MiSeq platform according to the standard protocols.

Raw fastq files were demultiplexed and quality-filtered using QIIME (version 1.17, GitHub, San Francisco, CA, USA)) with the following criteria: the 300 bp reads were truncated at any site receiving an average quality score <20 over a 10 bp sliding window, discarding the truncated reads that were shorter than 50 bp; exact barcode matching, 2 nucleotide mismatch in primer matching, reads containing ambiguous characters were removed; and, only sequences that overlap longer than 10 bp were assembled according to their overlap sequence. Reads that could not be assembled were discarded.

Operational taxonomic units (OTUs) were clustered with 97% similarity cutoff while using UPARSE (version 7.1 http://drive5.com/uparse/, Tiburon, CA, USA.) and chimeric sequences were identified and removed using UCHIME. RDP Classifier analyzed the taxonomy of each 16S rRNA gene sequence (http://rdp.cme.msu.edu/) against the silva (SSU115) 16S rRNA database using a confidence threshold of 70% [25]. The coverage percentage using Good’s method [26], the bias-corrected Chao richness estimator, and the Shannon diversity indices using the MOTHUR program (http://www.mothur.org, Ann Arbor, MI, USA) [27] were calculated.

### 2.6. Statistical Analysis

For all of the analyses, the individual hen was considered the experimental unit. Only data that were normally distributed and with equal variances were analyzed as a one-factor analysis of variance (ANOVA) using the Mixed procedure of SAS (SAS Inst. Inc., Cary, NC, USA). Data that were not normally distributed following log transformation or that had un-equal variances were subjected to nonparametric analysis using the Kruskal–Wallis test within the NPAR1WAY procedure of SAS. A *p* value of ≤0.05 was the level of signi**fi**cance for all tests. Tendencies were reported up to a *p* value of ≤0.15. Relative abundances are presented as means [28].

## 3. Results

### 3.1. Maize Grain and Diet Compositions

No major differences were observed between the BT maize or CC maize and the CT maize, and almost all of the values remained within the natural range of variation in maize varieties cited in the literature [29,30,31] (Table 1). The CT maize seemed to be a relative greater total phosphorus and lower crude protein than the transgenic maize lines, but the values were within the normal variability for maize varieties that were cited in the literature [31]. Amino acid contents were similar among the BT maize, CC maize, and CT maize ingredients. Proximate compositions and amino acid contents were also similar for the three maize-based diets (Table S1).

### 3.2. Ileal Histology

**Figure 1:**
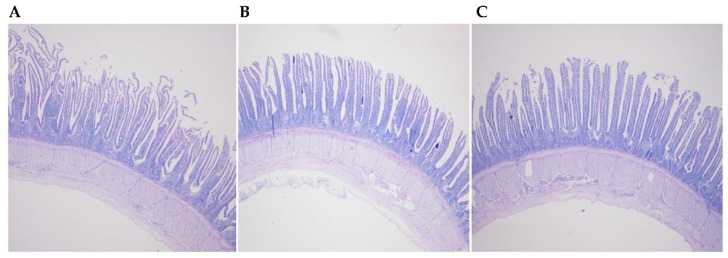
Histological examination of the ileum of laying hens fed the transgenic maize-based diet. (**A**) the nontransgenic near-isoline (CT) maize-based diet was fed to laying hens; (**B**) the transgenic *mCry1Ac* (BT) maize-based diet was fed to laying hens; and, (**C**) the transgenic *maroACC* (CC) maize-based diet was fed to laying hens.

Histological examination of the ileum did not indicate an effect of feeding the two transgenic maize lines (Figure 1). There were no statistically significant differences in villus height, crypt depth, and villus height/crypt depth in the ileum for birds that consumed the BT maize diet and CC maize diet for 12 weeks (Table 2). No significantly difference in goblet cell number/villus and goblet cell number/micrometer villus in the ileum were observed among the diet treatments.

### 3.3. Microbial Population Indices

**Figure 2:**
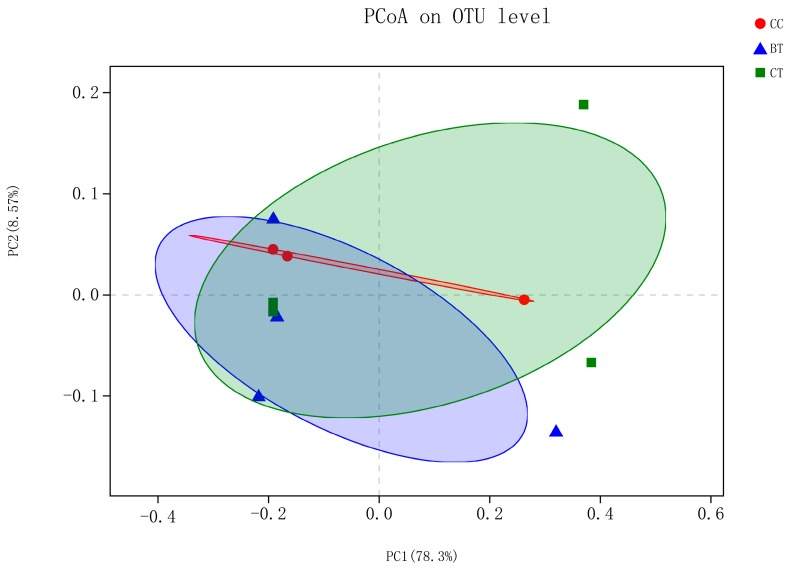
Unweighted bacterial beta diversity in the ileum of laying hens fed the transgenic maize-based diet. ■ The nontransgenic near-isoline (CT) maize-based diet was fed to laying hens; ▲ the transgenic *mCry1Ac* (BT) maize-based diet was fed to laying hens; ● the transgenic *maroACC* (CC) maize-based diet was fed to laying hens.

High-throughput sequencing of ileal samples from hens generated 16,188 sequences per bird of the V3–V4 region of the 16S rRNA gene. At the 97% similarity level, there were no differences in population indices, including Chao 1 richness estimation, Shannon diversity index, and Good’s coverage, among the treatments (Table 3). Shannon–Wiener curves showed similar levels of bacterial diversity among the diet treatments (Figure S1). Beta diversity analysis using the unweighted option did not reveal a split between the diet treatments (Figure 2).

### 3.4. The Relative Abundance of the Ileal Microbiota

**Figure 3:**
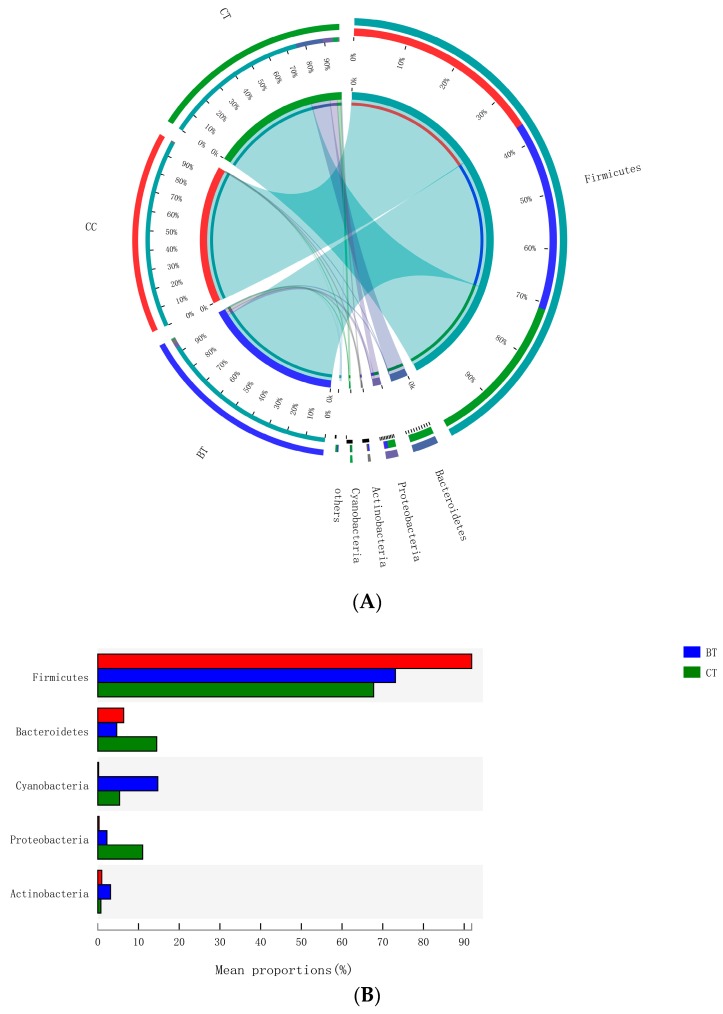
(**A**) Circos plot the relationship between the diet treatments and bacterial phyla. (**B**) Long-term effect of feeding the transgenic maize-based diet to laying hens on relative abundance of major ileal bacterial phyla. ■ The nontransgenic near-isoline (CT) maize-based diet, ■ the transgenic *mCry1Ac* (BT) maize-based diet were fed to laying hens, and ■ the transgenic *maroACC* (CC) maize-based diet.

A total of 17 different ileal bacterial phyla were detected. However, 99.0% of the sequence reads classified at the phylum level were derived from five phyla: *Firmicutes* (77.6% of total), *Bacteroidetes* (8.5%), *Cyanobacteria* (6.8%), *Proteobacteria* (4.5%), and *Actinobacteria* (1.6%), with the remaining 12 phyla accounting for only 1.0% of the sequence reads (Figure 3A). No significant differences were observed with respect to the relative abundances of bacterial phyla in the ileum of hens that were fed the BT or CC diets versus the CT diet (Figure 3B).

**Figure 4:**
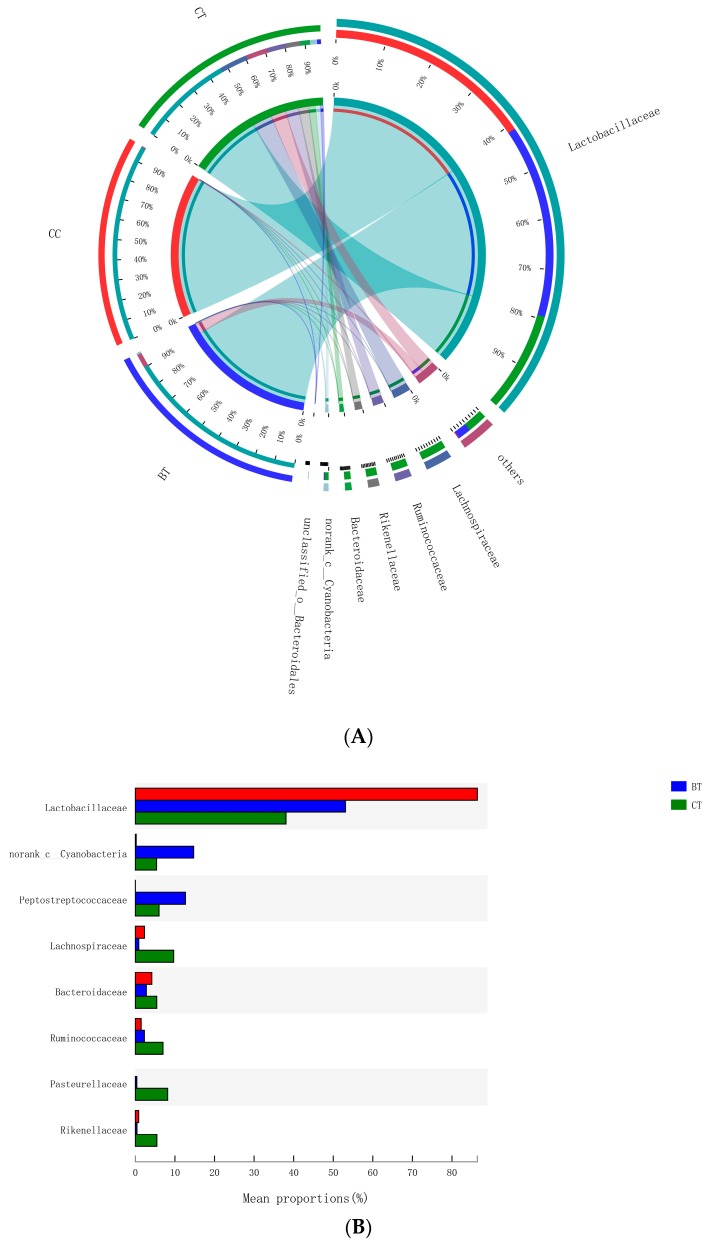
(**A**) Circos plot the relationship between the diet treatments and bacterial families. (**B**) Long-term effect of feeding the transgenic maize-based diet to laying hens on relative abundance of major ileal bacterial families. ■ The nontransgenic near-isoline (CT) maize-based diet, ■ the transgenic *mCry1Ac* (BT) maize-based diet were fed to laying hens, and ■ the transgenic *maroACC* (CC) maize-based diet.

**Figure 5:**
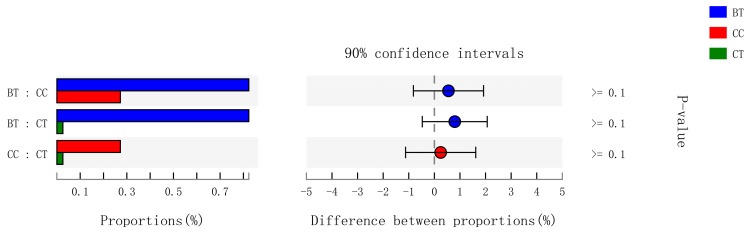
Kruskal–Wallis H test bar plot for *Bifidobacteriaceae* family. ■ The nontransgenic near-isoline (CT) maize-based diet, ■ the transgenic *mCry1Ac* (BT) maize-based diet were fed to laying hens, and ■ the transgenic *maroACC* (CC) maize-based diet. ● The relative abundance of *Bifidobacteriaceae* family in the BT diet subtracted by in the CC or CT diet, and ● the relative abundance of *Bifidobacteriaceae* family in the CC diet subtracted by in the CT diet.

**Figure 6:**
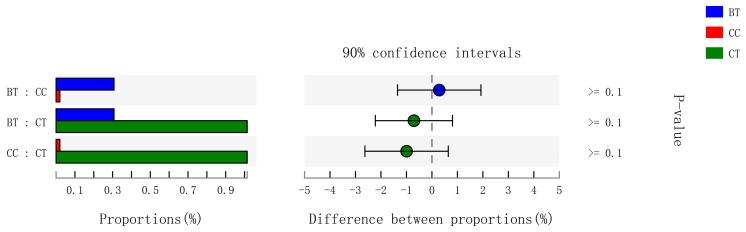
Kruskal–Wallis H test bar plot for *Enterobacteriaceae* family. ■ The nontransgenic near-isoline (CT) maize-based diet, ■ the transgenic *mCry1Ac* (BT) maize-based diet were fed to laying hens, and ■ the transgenic *maroACC* (CC) maize-based diet. ● The relative abundance of *Enterobacteriaceae* family in the BT diet subtracted by in the CC diet, and ● the relative abundance of *Enterobacteriaceae* family in the CT diet subtracted with in the BT or CC diet.

A total of 89 different bacterial families were detected in the hen ileum. The most abundant (89.2%) among the treatments were *Lactobacillaceae* (59.2%), *norank_Cyanobacteria* (6.8%), *Peptostreptococcaceae* (6.2%), *Lachnospiraceae* (4.3%), *Bacteroidaceae* (4.1%), *Ruminococcaceae* (3.6%), *Pasteurellaceae* (2.8%), and *Rikenellaceae* (2.2%) (Figure 4A). Few differences were observed among the diet treatments in the relative abundance of any of these major families (Figure 4B). There was a tendency (*p* = 0.109) for an increase in *Bifidobacteriaceae* abundance in the ileum of hens that were fed the CC maize-based diet (0.82%) and BT maize-based diet (0.27%) than hens fed the CT maize-based diet (0.03%; Figure 5). Birds that consumed the CC (0.02%) maize diet tended to have less abundance (*p* = 0.135) of *Enterobacteriaceae* in the ileum than those that consumed the CT maize diet (1.01%; Figure 6).

**Figure 7:**
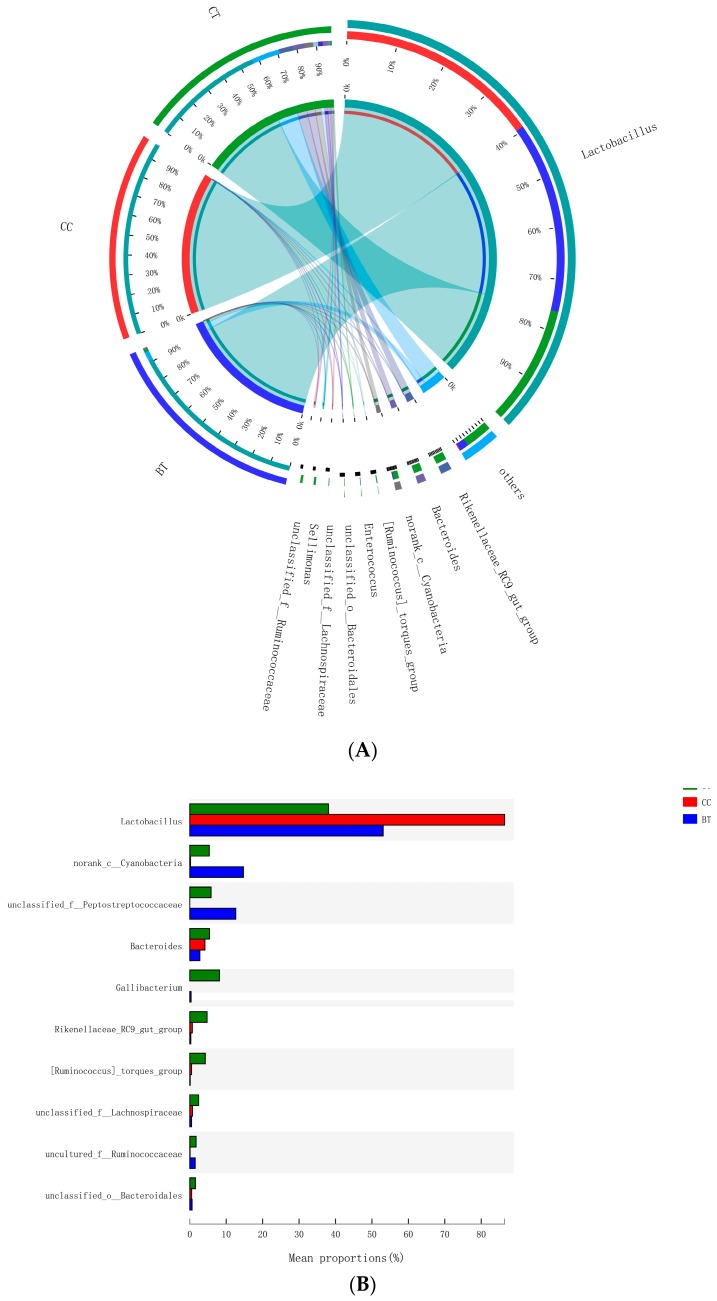
(**A**) Circos plot the relationship between the diet treatments and bacterial genera. (**B**) Long-term effect of feeding the transgenic maize-based diet to laying hens on relative abundance of major ileal bacterial genera. ■ The nontransgenic near-isoline (CT) maize-based diet, ■ the transgenic *mCry1Ac* (BT) maize-based diet were fed to laying hens, and ■ the transgenic *maroACC* (CC) maize-based diet.

**Figure 8:**
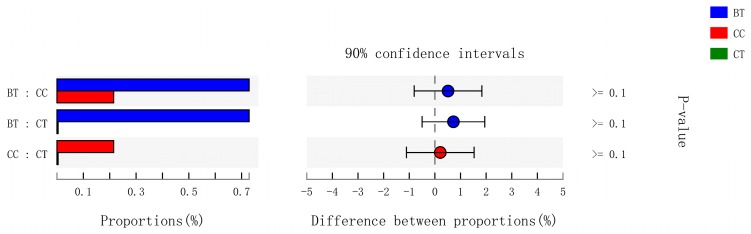
Kruskal-Wallis H test bar plot for *Bifidobacterium* genera. ■ The nontransgenic near-isoline (CT) maize-based diet, ■ the transgenic *mCry1Ac* (BT) maize-based diet were fed to laying hens, and ■ the transgenic *maroACC* (CC) maize-based diet. ● The relative abundance of *Bifidobacterium* genera in the BT diet subtracted by in the CC or CT diet, and ● the relative abundance of *Bifidobacterium* genera in the CC diet subtracted by in the CT diet.

A total of 199 genera were identified in the ileum of hens. The Figure 7A summarizes the 10 most abundant (85.8%) genera identified in the laying hen ileum, which included *Lactobacillus* (59.2%), *norank_Cyanobacteria* (6.8%), *unclassified_Peptostreptococcaceae* (6.2%), *Bacteroides* (4.1%), *Gallibacterium* (2.8%), *Rikenellaceae_RC9_gut_group* (1.9%), *[Ruminococcus]_torques_group* (1.6%), *unclassified_Lachnospiraceae* (1.2%), and *uncultured_Ruminococcaceae* (1.1%). There were no significant differences among treatments in the relative abundance of any of these major genera (Figure 7B). However, birds that consumed the BT (0.73%) and CC (0.21%) maize diet tended to have greater abundance (*p* = 0.114) of *Bifidobacterium* in the ileum than those that consumed the CT maize diet (0.004%; Figure 8).

## 4. Discussion

The intestinal microbiota plays a profound role in health and extensive research has been dedicated to the strong interplay between intestinal microbiota and host disease [2,3]. To date, research investigating the effect of feeding transgenic crops on gastrointestinal bacterial communities has been limited to studies in large intestinal and fecal microbiota [11,12,13,14,15,16], whereas the microbiota in the small intestine also has a very important effect on immune response, metabolic, and endocrine functions [17]. To our knowledge, the present study is the first to employ deep sequencing to characterize ileal microbiota composition fed the transgenic maize-based diets.

This deep 16S rRNA gene-sequencing approach detected different bacterial phyla in the ileal content samples of birds, with *Firmicutes*, *Bacteroidetes*, *Cyanobacteria,* and *Proteobacteria* dominating. The relative distributions agree with that previously observed in the ileum of hens using 16S rRNA gene sequencing [32,33]. Similarly, Xu et al. [32] detected bacterial phyla in the ileum of chickens, but found that *Firmicutes* (average relative abundance >75%) dominated, followed by *Proteobacteria*, *Cyanobacteria,* and *Bacteroidetes*. Sequence-based compositional analysis of the ileal microbiota revealed no significant differences in the relative abundance of bacterial phyla between the transgenic maize and isogenic maize-fed hens, indicating that the BT maize and the CC maize are well tolerated by the host and intestinal microbiota at the phylum level. Similarly, no effect of feeding BT maize to weanling pigs for 31 days [15] and to finishing pigs for 110 days [34] were found in the relative abundance of cecal bacterial phyla.

Dominant bacterial families, including *Lactobacillaceae* (59.2%), *norank_Cyanobacteria*, *Peptostreptococcaceae*, *Lachnospiraceae*, and *Bacteroidaceae* were detected in the present study. Kollarcikova et al. [35] also detected bacterial family in the ileum of hens and found that *Lactobacillaceae* dominated in adult hens and was followed by *Peptostreptococcaceae*. Sequence-based compositional analysis of the ileal microbiota revealed no significant differences in the relative abundance of the major bacterial family between the transgenic maize and isogenic maize-fed hens, indicating that the CC maize and the BT maize are well tolerated by the host and ileal microbiota at the family level. Similarly, no effect of feeding BT maize to sow for 110 days and to finishing pigs for 110 days [16,34] were found in relative abundance of cecal bacterial families.

The only statistically difference with the ileal microbiota was observed that the abundance of *Bifidobacteriaceae* was a tendency to be greater in the ileal samples of birds that were fed the transgenic maize-based diets than those hens fed the CT maize-based diet in the present study. However, the different tendency is not likely to have a detrimental effect on the host. Similarly, Buzoianu et al. [15] detected that pigs consumed the BT maize diet had higher cecal abundance of *Bifidobacteriaceae* (0.04 versus 0%) than those that consumed the isogenic maize diet. However, these differences are unlikely to have an adverse effect on the host. In fact, family *Bifidobacteriaceae* is considered to be the most important beneficial microbes in the gut [36,37]. Birds that consumed the CC (0.02%) maize diet tended to have less abundance of *Enterobacteriaceae* in the ileum than those that consumed the CT maize diet (1.01%), which agrees with the observation that fecal *Enterobacteriaceae* in the transgenic maize treatment was numerically (not significantly) is less than in the non-transgenic maize treatment [15]. Buzoianu et al. [16] also found that pigs fed the GM maize for 115 days had lower ileal *Enterobacteriaceae* counts than pigs that were fed the non-GM maize. *Enterobacteriaceae* in the phylum *Proteobacteria,* many of the more familiar pathogens [38,39], such as *Salmonella* [40,41] and *Escherichia coli* [42,43], plays a critical role in the enteric disease of humans and animals. In the present study, the difference in ileal *Bifidobacteriaceae* and *Enterobacteriaceae* abundance was not associated with any effects on intestinal morphology and host health.

In the present study, dominant bacterial genera, with *Lactobacillus*, *norank_Cyanobacteria*, *unclassified_Peptostreptococcaceae*, *Bacteroides*, *Gallibacterium*, *Rikenellaceae_RC9_gut_group*, *[Ruminococcus]_torques_group*, *unclassified_Lachnospiraceae*, and *uncultured_Ruminococcaceae* were detected. Lactobacillaceae, with an average relative abundance >59%, dominated in the ileum of hens, which agreed with previous studies [44]. Sequence-based compositional analysis of the ileal microbiota revealed no significant differences in relative abundance of any of major genera between the transgenic maize and isogenic maize-fed hens. Similarly, no effect of feeding BT maize to pigs [15,16,34] was found in the relative abundance of major genera. In the present study, a relative abundance of Bifidobacterium belongs within the *Bifidobacteriaceae* family tended to be greater in the ileal samples of hens that were fed the transgenic maize-based diet than those fed the non-transgenic maize-based diet. Similarly, Buzoianu et al. [15] detected pigs that consumed the BT maize diet had higher cecal abundance of Bifidobacterium (0.04 versus 0%) than those that consumed the isogenic maize diet. Li et al. [45] also found that fecal Bifidobacterium abundance (2.17 versus 0.13%) was greater in the female rats that were fed the transgenic maize carrying the Cry1Ab and EPSPS genes than those fed the non-transgenic maize. The Bifidobacterium are not only considered to be the most important beneficial microbes in the human gut [36,37], but also considered as probiotics in the chicken intestinal tract [46,47]. The enrichment of Bifidobacterium in the ileum of birds indicated that feeding transgenic maize-diet might facilitate the growth of potentially beneficial bacteria in the gut. The role of Bifidobacterium in the chicken small intestine has not yet been fully elucidated, when considering that they are not numerically dominant. Histological examination of intestinal tissue from these hens did not reveal any signs of intestinal damage or inflammation. Therefore, although statistically tendency, the difference in the ileal abundance of Bifidobacterium observed in the present study is not believed to be of biological significance or to have a negative impact on animal health.

## 5. Conclusions

In summary, the results from the present study indicate that dietary BT maize and CC maize are well tolerated at the level of the ileal microbiota following 12 weeks of exposure in laying hens. Few effects were observed within the ileal microbial community structure of hens following long term exposure to transgenic maize. The low abundance and frequency of detection of some taxa are not believed to be of major biological importance and they were not associated with any adverse health effects. The results may provide a scientific basis for evaluating the biosafety of long-term feeding BT maize and CC maize in terms of the ileal microbiome.

## Figures and Tables

**Table 1:** Chemical and amino acid analysis of maize (as fed basis).

Nutrient, g/kg	Maize ^†^			Normal Value (%) (Reference)
	CT	BT	CC	
Dry matter	866.9	867.4	865.8	856–906 [31]
Crude protein	67	74	77	60–127 [31]
Ether extract	31	30	32	17–60 [29]; 31–58 [31]
Ash	11	11	11	6–60 [29]; 13–15 [31]
Starch	663	654	669	256–754 [29]; 546–699 [31]
Neutral detergent fiber	98	101	104	110–147 [30]; 83–119 [31]
Acid detergent fiber	18	17	17	36–48 [30]; 30–43 [31]
Calcium	0.1	0.1	0.1	0.03–1.0 [31]
Total phosphorus	2.1	1.6	1.6	2.3–7.5 [31]
Gross energy, kcal/kg	3833	3848	3888	-
Essential amino acids				
Arginine	3.5	3.4	3.7	2.2–6.4 [31]
Histidine	2.8	3.0	3.1	1.5–3.8 [31]
Isoleucine	2.9	2.6	2.9	2.2–7.1 [31]
Leucine	9.0	9.0	10.1	7.9–24.1 [31]
Lysine	2.5	2.5	2.6	0.5–5.5 [31]
Methionine	1.2	1.2	1.2	1.0–4.6 [31]
Phenylalanine	4.2	4.1	4.6	2.9–6.4 [31]
Threonine	3.1	3.0	3.3	2.7–5.8 [31]
Valine	4.6	4.6	4.7	2.1–8.5 [31]
Non-essential amino acids				
Alanine	5.8	5.8	6.5	5.6–10.4 [31]
Aspartic acid	5.0	4.9	5.4	4.8–8.5 [31]
Cysteine	1.5	1.5	1.5	0.8–3.2 [31]
Glutamic acid	14.2	14.5	15.9	12.5–25.8 [31]
Glycine	3.0	2.9	3.3	2.6–4.9 [31]
Proline	7.0	6.9	8.1	6.3–11.6 [31]
Serine	3.9	3.9	4.3	3.5–9.1 [31]
Tyrosine	4.1	4.0	4.3	1.2–7.9 [31]

^†^ CT = nontransgenic near-isoline maize, BT = transgenic maize produced by the insertion of the *mCry1Ac* gene derived from *Bacillus thuringiensis* strain, and CC = transgenic maize produced by the insertion of the *maroACC* gene derived from *Agrobacterium tumefaciens* strain.

**Table 2:** Long-term effect of feeding genetically modified (GM) maize to laying hens on ileal histology.

Item	Diet ^†^			SEM	*p*-Value ^§^
	CT	BT	CC		
Villus height (μm)	688	704	593	32.8	NS
Crypt depth (μm)	151	127	132	5.5	NS
Villus height/crypt depth	4.56	5.33	4.89	0.538	NS
Goblet cells/villus	40.1	63.4	62.8	5.50	NS
Goblet cells/μm villus	59.8	75.4	82.3	5.23	NS

^†^ CT = nontransgenic near-isoline maize, BT = transgenic maize produced by the insertion of the *mCry1Ac* gene derived from *Bacillus thuringiensis* strain, and CC = transgenic maize produced by the insertion of the *maroACC* gene derived from *Agrobacterium tumefaciens* strain. ^§^ NS: Mean values were no significantly different among 3 diet treatments (*p* < 0.05).

**Table 3:** Long-term effect of feeding transgenic maize on bacterial diversity in laying hens.

Microbiota Source and Diversity Measure	Diet ^†^			
	CT	BT	CC	*p*-Value ^§^
Chao 1 richness estimation	216	171	153	NS
Shannon diversity index	3.1	1.9	2.1	NS
Good’s coverage	0.997	0.998	0.998	NS

^†^ CT = nontransgenic near-isoline maize, BT = transgenic maize produced by the insertion of the *mCry1Ac* gene derived from *Bacillus thuringiensis* strain, and CC = transgenic maize produced by the insertion of the *maroACC* gene derived from *Agrobacterium tumefaciens* strain. ^§^ NS: Mean values were no significantly different among 3 diet treatments (*p* < 0.05).

## Supplementary Materials

The following are available online https://www.mdpi.com/2076-2607/7/3/92/s1, Table S1: Ingredient and analyzed nutrient composition (as-fed basis) of diets. Figure S1. Shannon-Wiener curves for ilea bacteria of laying hens fed the transgenic corn-based diet.
