# Supplementary material for: The Chronic Effect of Transgenic Maize Line with mCry1Ac or maroACC Gene on Ileal Microbiota Using a Hen Model
Source: Microorganisms. 2019 Mar 24;7(3):92. doi: 10.3390/microorganisms7030092 (PMC6463162; doi:10.3390/microorganisms7030092)
Supplement: Supplementary file 1 [file microorganisms-07-00092-s001.zip › Table S1.pdf]

Supplementary Table

Table S1. Ingredient and analyzed nutrient composition (as-fed basis) of diets.

Item	Diet[a]		
	CT	BT	CC
Ingredient, g/kg			
Corn	617.0	617.0	617.0
Nontransgenic soybean meal	262.0	262.0	262.0
Limestone	81.0	81.0	81.0
Dicalcium phosphate	15.1	15.1	15.1
Non transgenic soybean oil	10.0	10.0	10.0
Methionine	3.0	3.0	3.0
Salt	1.0	1.0	1.0
Choline chloride	1.0	1.0	1.0
Premix[b]	10.0	10.0	10.0
Analyzed nutrient composition			
Dry matter	922.2	912.2	907.3
Crude protein	168	167	165
Ether extract	32	32	33
Ash	135	131	136
Starch	382	383	382
Neutral detergent fiber	65	69	69
Acid detergent fiber	23	23	21
Calcium	41.7	40.3	41.4
Total phosphorus	5.3	4.6	4.5
Gross energy, kcal/kg	3,584	3,630	3,574
Indispensable amino acid			
Arginine	11	11	11
Histidine	5.0	5.2	5.0
Isoleucine	6.8	7.2	6.5
Leucine	14.3	14.0	13.9
Lysine	9.0	9.1	8.7
Methionine	3.6	3.1	2.9
Phenylalanine	8.3	8.5	8.2
Threonine	6.4	6.6	6.5
Valine	7.9	7.9	7.5
Dispensable amino acid			
Alanine	8.4	8.9	8.8
Aspartic acid	16.2	16.4	16.1
Cysteine	2.4	2.3	2.3
Glutamic acid	28.8	29.8	29.6
Glycine	6.8	7.0	6.7
Proline	9.7	10.0	10.2
Serine	7.8	8.5	8.5
Tyrosine	6.5	6.8	6.7

[a]CT: nontransgenic near-isoline corn, BT: transgenic corn produced by the insertion of the *mCry1Ac* gene derived from *Bacillus thuringiensis* strain, and CC: transgenic corn produced by the insertion of the *maroACC* gene derived from *Agrobacterium tumefaciens* strain.

[b]Provided per kilogram of diet: vitamin A, 12,500 IU; vitamin D_3, 4,125 IU; vitamin E, 15 IU; vitamin K, 2 mg; thiamine, 1 mg; riboflavin, 8.5 mg; calcium pantothenate, 50 mg; niacin, 32.5 mg; pyridoxine, 8 mg; biotin, 2 mg; folic acid, 5 mg; vitamin B_{12}, 5 mg; manganese, 80 mg; iodine, 1 mg; iron, 60 mg; copper, 8 mg; zinc, 80 mg; selenium, 0.3 mg.
